# Supplementary material for: Rete ridges form via evolutionarily distinct mechanisms in mammalian skin
Source: Nature. 2026 Feb 4;651(8104):135–45. doi: 10.1038/s41586-025-10055-5 (PMC12959975; doi:10.1038/s41586-025-10055-5)
Supplement: Supplementary file 1 — Supplementary Figs. 1–13. [file 41586_2025_10055_MOESM1_ESM.pdf]

nature portfolio

https://doi.org/10.1038/s41586-025-10055-5

Supplementary information

Rete ridges form via evolutionarily distinct mechanisms in mammalian skin

In the format provided by the authors and unedited

Supplementary Figures

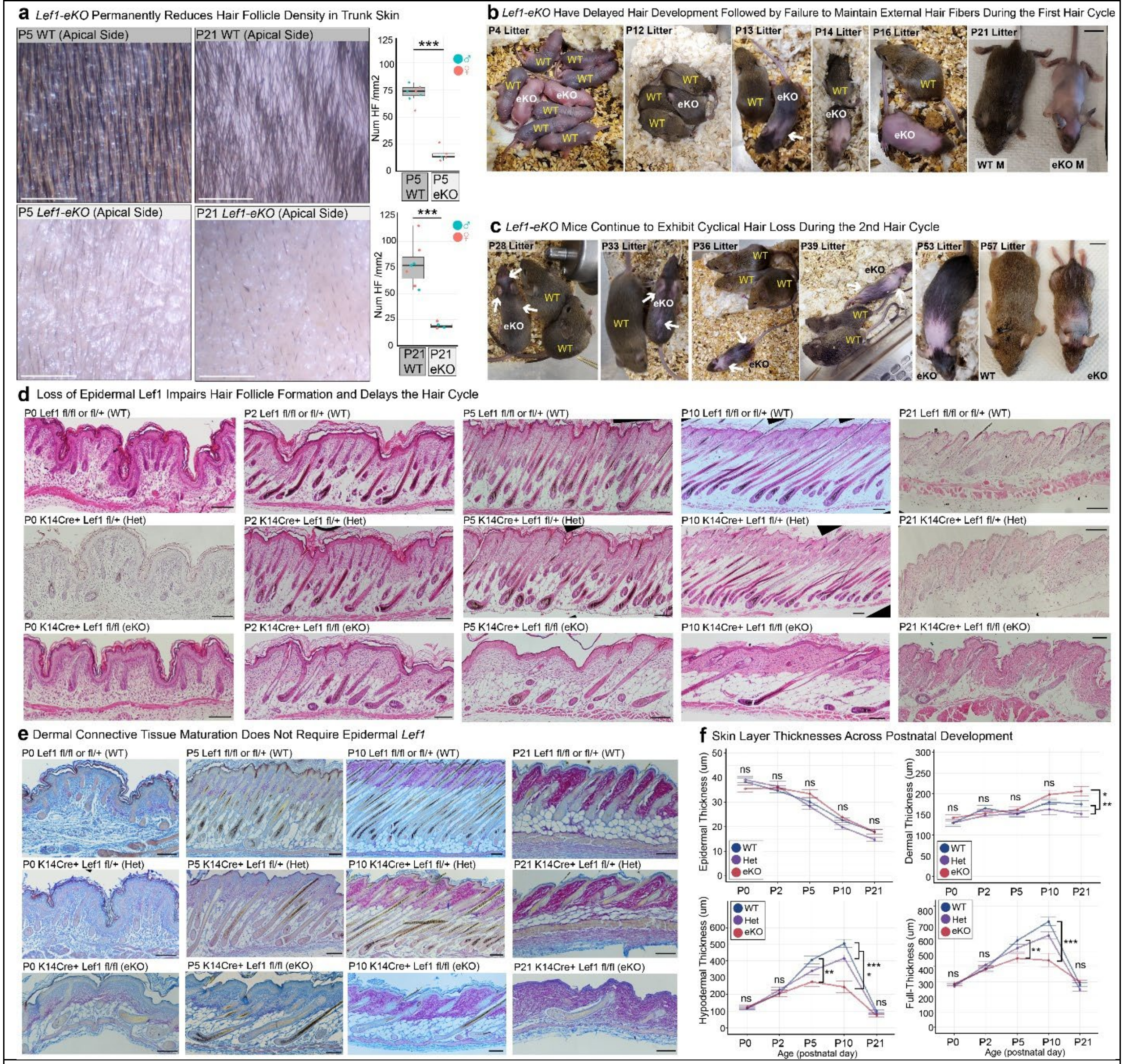

Supplemental Figure 1: Epidermal LEF1 Regulates Hair Density and Genetic Reduction of Hair Density Does Not Increase Epidermal Thickness. (a) Dissecting microscope images (left) of the apical surface of the epidermis to visualize hair follicle density between WT phenotype (top) and *K14-Cre;Lef1$^{fl/fl}$* (*Lef1-eKO/*eKO, bottom) at P5 (n=8, 5) and P21 (n=7, 4) mice from 2 separate litters per timepoint. Scale bar represents 1mm. Quantification (right) of P5 and P21 WT vs eKO hair density /mm^2. Individual datapoints colored by sex (teal male, salmon female). *** = p-value < 0.001 from t-test, P5 p-value = 5.639e-08, P21 p-value = 0.0002511 . **(b-c)** images of the coats of *Lef1-eKO* mice during the (b) first and (c) second hair cycle. eKO mouse labeled in P12-16 timepoints was the same biological replicate paired with different WT phenotype littermates in each picture due to lack of identifying markings. The same eKO mouse and WT littermates continued to be aged and assessed from P28-P57 in (c). P21 males in (b) were collected from the same litter as the representative females in Figure 2f. Phenotypes observed in (b) were consistent across more than five separate litters,

and (c) across more than three separate litters. Scale bar represents 1cm. WT=wild-type phenotype mouse, eKO = *Lef1-eKO*. **(d)** representative H&E stains at P0, P2, P5, P10, and P21 for WT ($Lef1^{fl/fl}$ or $Lef1^{fl/+}$), *K14-Cre;Lef1*$^{fl/+}$ (*Het*), and *Lef1-eKO* genotyped mice. **(e)** representative Herovici stains at P0, P5, P10, and P21 for WT, Het, and eKO mice. (d-e) scale bars represent 100um. **(f)** Line plots of Epidermal Thickness (top left), Dermal Thickness (top right), Hypodermal Thickness (bottom left), and Full-thickness (bottom right) in um from WT, Het, and eKO at P0 (n=24, 9, 13), P2 (n=3, 3, 3), P5 (n=27, 16, 14), P10 (n=21, 5, 8), and P21 (n=32, 11, 19). All time points represent at least 3 separate litters except for P2, which was 1 litter. One-way ANOVA plus Tukey HSD, ns=p-values >=0.05, *=p-value <0.05, **=p-value <- 0.01, ***=p-value <0.001. See Source Data for complete list of comparisons and exact p-values. Error bars represent SEM.

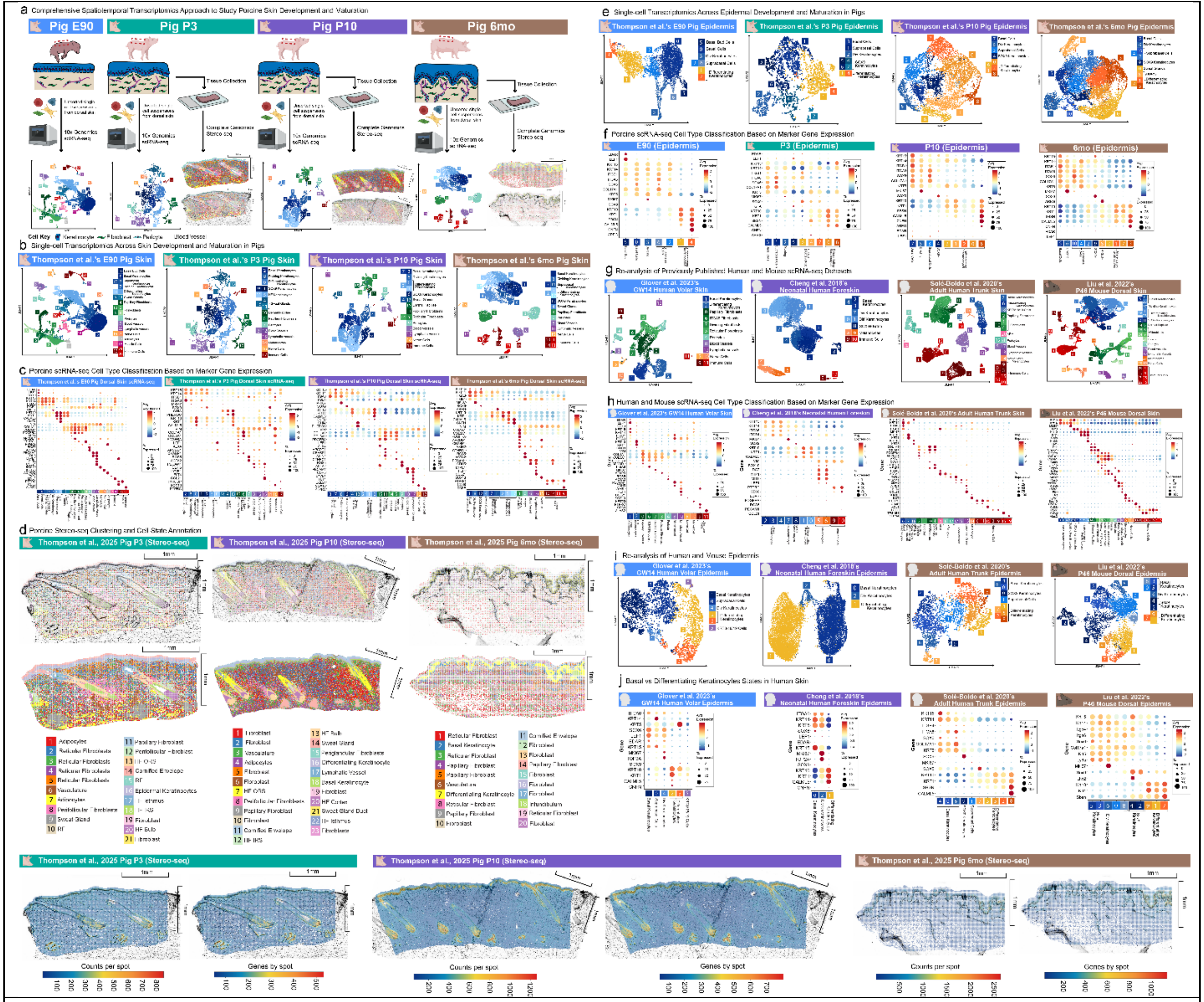

Supplemental Figure 2: Massively Parallel Multi-Species Spatiotemporal Transcriptomics Analysis of Pig and Re-analysis of Human and Mouse Skin. (a) Schematic of spatiotemporal transcriptomics approach to investigate porcine rete ridge formation. **(b)** E90, P3, P10, and 6mo pig scRNA-seq full datasets visualized using UMAP. Clusters are colored by their respective cell lineage based on SLM algorithm. **(c)** Dotplots depicting cluster-level expression of DEGs/canonical markers used to annotate each cluster's cell type. **(d)** P3, P10, and 6mo Stereo-seq bin size 20 (10um spot) datasets visualizing Leiden-clustering of spots over the tissue mask. Scale bars indicate 1mm. (bottom, wide) QC spatial scatter plots depicting number of reads per spot and number of genes per spot (bin size 20) for P3, P10, and 6mo Stereo-seq datasets. **(e)** E90, P3, P10, and 6mo pig scRNA-seq epidermal subsets visualized using UMAP based on SLM algorithm. **(f)** Dotplots depicting cluster-level expression of canonical epidermal heterogeneity markers used to annotate each cluster's cell state. **(g)** Re-analysis of previously published human full scRNA-seq datasets visualized using UMAP and the SLM algorithm: Glover et al. 2023's GW14 Human Volar Skin, Cheng et al. 2018's Neonatal Human Foreskin, Solé-Boldo et al. 2020's Adult Human Trunk Skin (left) and Liu et al. 2022's Adult Mouse Back Skin (right). **(h)** Dotplots depicting cluster-level expression of DEGs/canonical markers used to annotate each cluster's cell type. **(i)** Re-analyzed human scRNA-seq epidermal subsets visualized using UMAP (left) and adult mouse scRNA-seq epidermal subset visualized using UMAP. **(j)** Dotplots depicting cluster-level expression of canonical markers used to annotate each cluster's cell state.

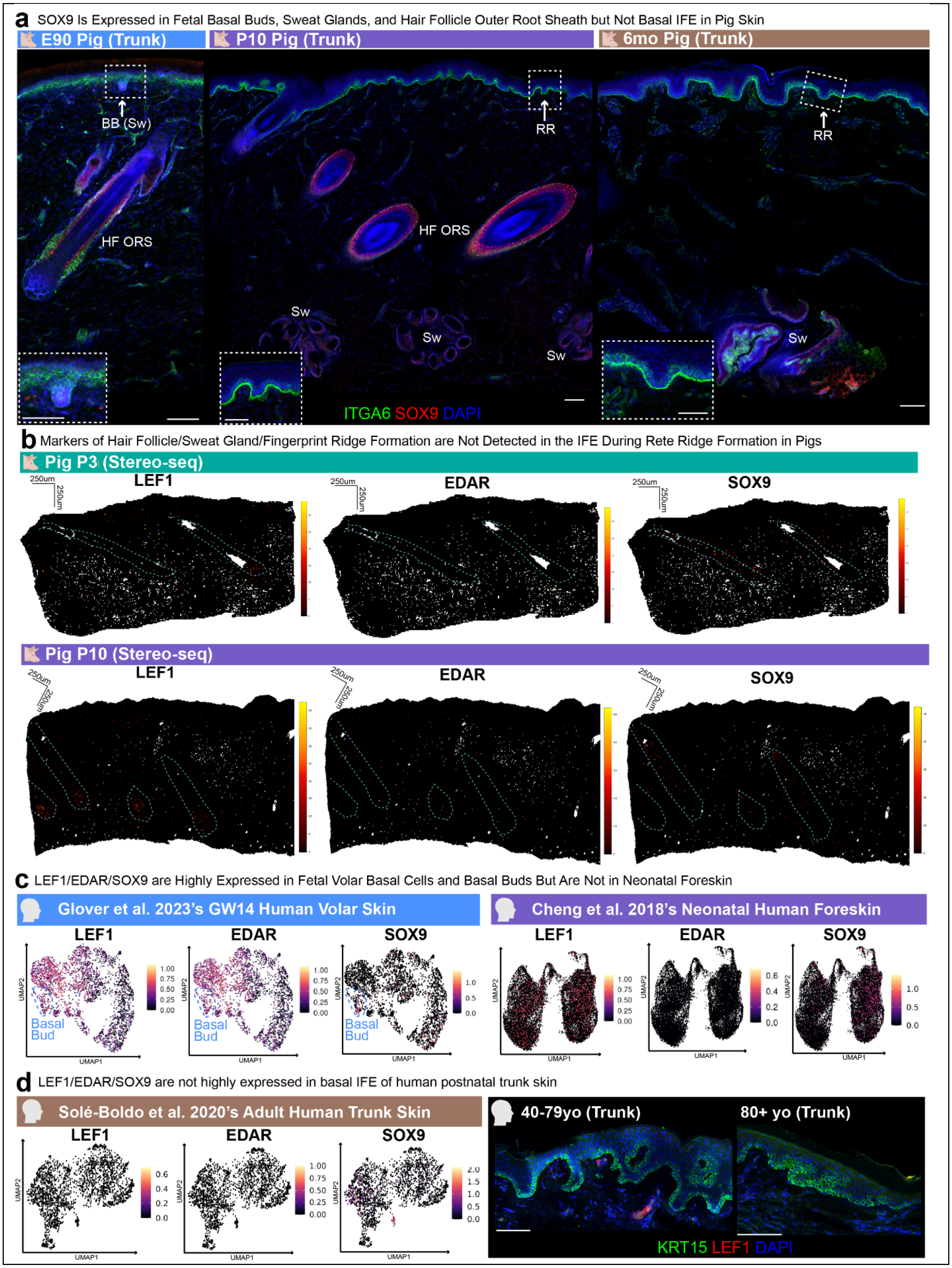
a SOX9 Is Expressed in Fetal Basal Buds, Sweat Glands, and Hair Follicle Outer Root Sheath but Not Basal IFE in Pig Skin
E90 Pig (Trunk)
P10 Pig (Trunk)
6mo Pig (Trunk)
BB (Sw)
HF ORS
RR
HF ORS
Sw
Sw
Sw
RR
Sw
ITGA6 SOX9 DAPI
b Markers of Hair Follicle/Sweat Gland/Fingerprint Ridge Formation are Not Detected in the IFE During Rete Ridge Formation in Pigs
Pig P3 (Stereo-seq)
LEF1
EDAR
SOX9
250um
Pig P10 (Stereo-seq)
LEF1
EDAR
SOX9
c LEF1/EDAR/SOX9 are Highly Expressed in Fetal Volar Basal Cells and Basal Buds But Are Not in Neonatal Foreskin
Glover et al. 2023's GW14 Human Volar Skin
Cheng et al. 2018's Neonatal Human Foreskin
LEF1
EDAR
SOX9
Basal Bud
UMAP1
UMAP2
d LEF1/EDAR/SOX9 are not highly expressed in basal IFE of human postnatal trunk skin
Solé-Boldo et al. 2020's Adult Human Trunk Skin
LEF1
EDAR
SOX9
40-79yo (Trunk)
80+ yo (Trunk)
KRT15 LEF1 DAPI

Supplemental Figure 3: Placode Markers Are Not Expressed During Rete Ridge Formation and Maturation in Pigs and Humans. (a) Representative immunostains from E90 (n=3), P10 (n=2), and 6mo (n=3) pig skin for ITGA6/SOX9. Zoom-out scale bars represents 100um. Dashed box indicates a region of zoom-in, with scale bar of 50um. BB= basal bud (sweat gland placode), Sw = sweat gland, HF ORS=hair follicle outer root sheath, RR=rete ridge. **(b)** P3 and P10 Stereo-seq visualization of LEF1, EDAR, and SOX9 spatial expression. Scale bars indicate 250um. Dashed teal outline represents shape of hair follicle in the tissue mask. White spots within the tissue mask indicate bin20 spots which did not pass QC and were filtered out. **(c)** FeaturePlot representations of expression of LEF1, EDAR, and SOX9 in Glover et al. 2023's GW14 Human fetal volar skin and Cheng et al. 2018's Neonatal Human Foreskin scRNA-seq epidermal subsets. The LEF1+/EDAR+/SOX9+ Basal Bud cells at GW14 are circled in blue. **(d)** FeaturePlot representations of expression of LEF1, EDAR, and SOX9 in Solé-Boldo et al. 2020's Adult Human Trunk Skin scRNA-seq epidermal subset. Representative immunostains of KRT15/LEF1 from adult trunk skin, aged 40-79yo (n=3) or 80+ yo (n=1). Scale bars represent 100um.

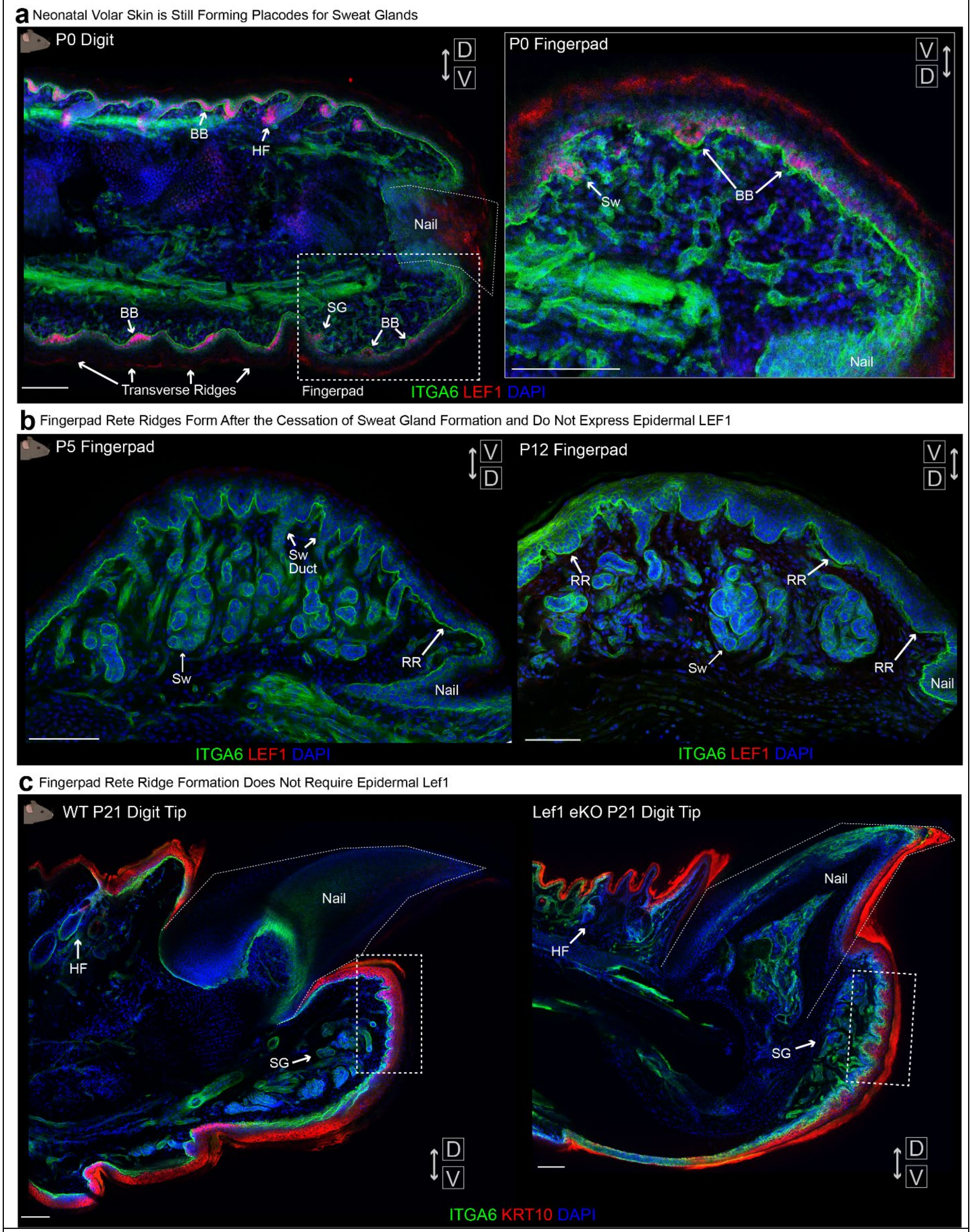

Supplemental Figure 4: Murine Fingerpads Form Rete Ridges and Do Not Require Epidermal LEF1. (a) Representative immunostains for Itga6/Lef1 in P0 digit (left, n=3) and fingerpad zoom-in (right). Digital volar transverse ridges are indicated with arrows. The Zoom-in is uniformly brightened relative to the zoom-out for improved readability. **(b)** Representative

immunostains for Itga6/Lef1 in P5 (left, n=3) and P12 (right, n=3) fingerpads. **(c)** Representative immunostains for Itga6/Krt10 P21 WT (n=3) and *Lef1-eKO* (n=3) digit tips. Dashed boxes at P21 depict the zoom-in region displayed in Fig. 3d. Scale bars represent 100um. BB=basal bud (placode for HF on dorsal, placode for sweat gland on ventral), HF=hair follicle, Sw=sweat gland, D and V indicate dorsal-ventral orientation.

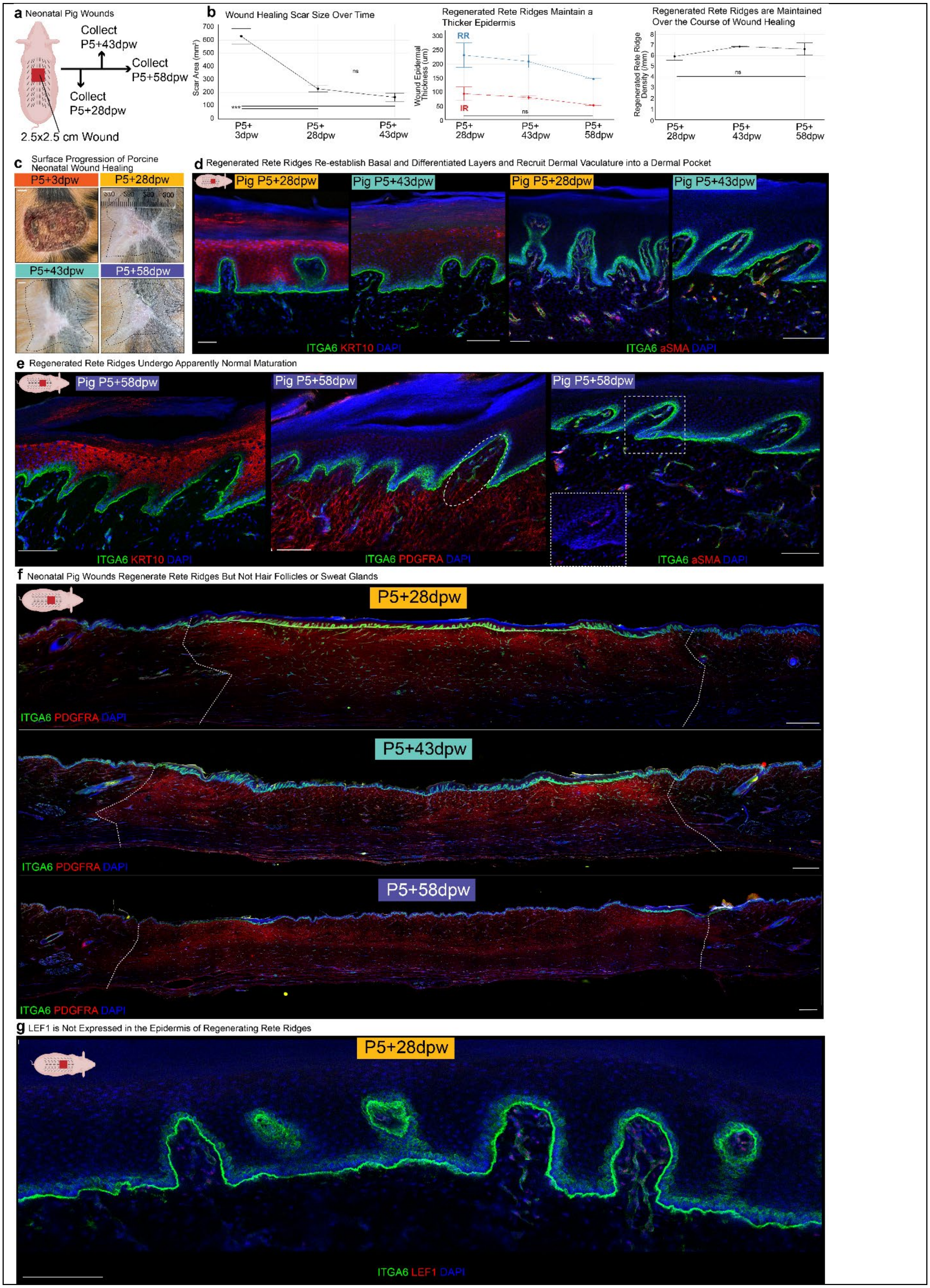
a Neonatal Pig Wounds
Collect P5+43dpw
Collect P5+58dpw
Collect P5+28dpw
2.5x2.5 cm Wound
b Wound Healing Scar Size Over Time
Scar Area (mm²)
P5+ 3dpw
P5+ 28dpw
P5+ 43dpw
ns
***
Regenerated Rete Ridges Maintain a Thicker Epidermis
Wound Epidermal Thickness (um)
RR
IR
ns
P5+ 28dpw
P5+ 43dpw
P5+ 58dpw
Regenerated Rete Ridges are Maintained Over the Course of Wound Healing
Regenerated Rete Ridge Density (/mm)
ns
c Surface Progression of Porcine Neonatal Wound Healing
P5+3dpw
P5+28dpw
P5+43dpw
P5+58dpw
d Regenerated Rete Ridges Re-establish Basal and Differentiated Layers and Recruit Dermal Vaculature into a Dermal Pocket
Pig P5+28dpw
Pig P5+43dpw
Pig P5+28dpw
Pig P5+43dpw
ITGA6 KRT10 DAPI
ITGA6 aSMA DAPI
e Regenerated Rete Ridges Undergo Apparently Normal Maturation
Pig P5+58dpw
Pig P5+58dpw
Pig P5+58dpw
ITGA6 KRT10 DAPI
ITGA6 PDGFRA DAPI
ITGA6 aSMA DAPI
f Neonatal Pig Wounds Regenerate Rete Ridges But Not Hair Follicles or Sweat Glands
P5+28dpw
ITGA6 PDGFRA DAPI
P5+43dpw
ITGA6 PDGFRA DAPI
P5+58dpw
ITGA6 PDGFRA DAPI
g LEF1 is Not Expressed in the Epidermis of Regenerating Rete Ridges
P5+28dpw
ITGA6 LEF1 DAPI

Supplemental Figure 5: Full-thickness Neonatal Pig Wounds Can Regenerate Rete Ridges. **(a)** Experimental design. Wounding cohort (n=7, same litter), collected at 28dpw (n=2), 43dpw (n=2), 58dpw (n=3). **(b, left)** Quantification of scar surface area (mm^2) at 3dpw (n=7), 28dpw (n=7), 43dpw (n=5), and 58dpw (n=3). One-way ANOVA with post-hoc Tukey's HSD, *** p-values < 0.001, ns p-value > 0.05. **(b, center/right)** Quantification of histology from 28 (n=2), 43 (n=2), and 58dpw (n=3) for **(center)** regenerated rete ridge and inter-ridge thickness (um) and **(right)** regenerated rete ridge density (/mm). Error bars represent SEM. One-way ANOVA, ns p-value > 0.05. See Source Data for complete list of comparisons and exact p-values for (b). **(c)** representative images of wound site at 3dpw (n=7), 28dpw (n=7), 43dpw (n=5), and 58dpw (n=3). Scale bar represents 5mm. **(d)** Representative immunostains from center of whole-wound 60um cross sections for ITGA6/KRT10 and ITGA6/aSMA at 28dpw (n=2) or 43dpw (n=2), imaged on a Leica SP8. **(e)** Representative immunostains of center of whole-wound 60um cross sections for ITGA6/KRT10 (n=3), ITGA6/PDGFRA (n=3), and ITGA6/aSMA (n=3), at 58dpw imaged on a Leica SP8. **(f)** Representative immunostains of entire whole-wound 60um cross sections for ITGA6/PDGFRA at 28dpw (n=2), 43dpw (n=2), and 58dpw (n=3), imaged on a Leica DMI8. Dashed line indicates the edges of the wound bed. Scale bars indicate 1 mm. **(g)** Representative immunostain of regenerating rete ridges at 28dpw for ITGA6/LEF1 (n=2). (d-e, g) Scale bars represent 100 um.

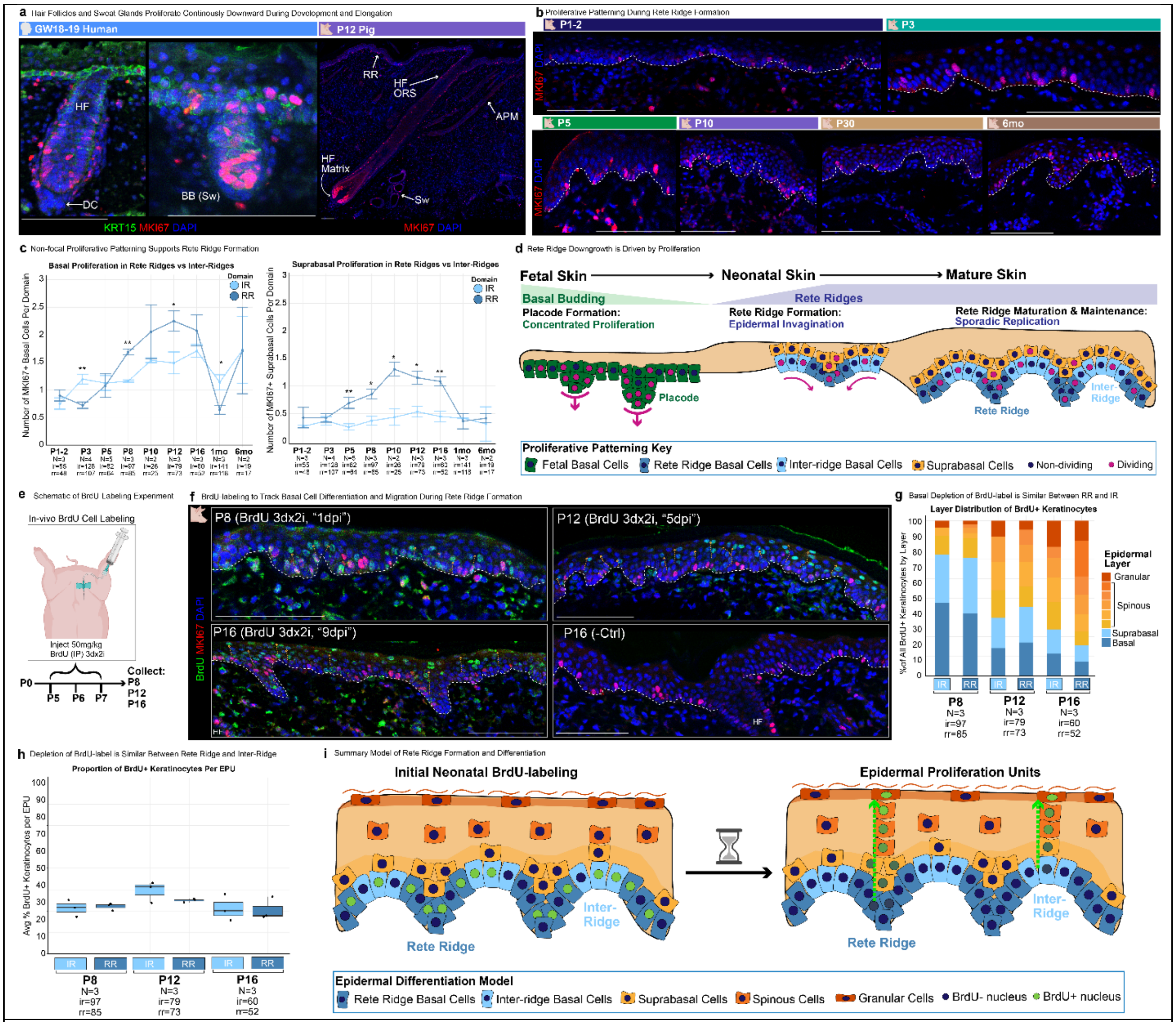

Supplemental Figure 6: Proliferation and Differentiation Support Rete Ridge Formation. **(a)** Representative immunostains of developing human hair follicles and sweat glands at GW18-19 (left, n=3) and mature porcine hair follicles and sweat glands at P12 (right, n=3) stained for MKI67. **(b)** Representative immunostains of MKI67 throughout rete ridge formation and maturation in pig back skin. **(c)** Quantification of basal (left) and suprabasal (right) proliferation between rete ridge and inter-ridge regions across postnatal porcine skin development. N=biological replicates, ir=number of inter-ridge regions, rr=number of rete ridge regions quantified and presented on the graph beneath the respective timepoint. Error bars represent SEM. ** = p-value < 0.01, * = p-value < 0.05, ns=not significant, p-value > 0.05 from t-test. From youngest to oldest, RR vs IR (basal): p-values = 0.3634, 0.005944, 0.8622, 0.004305, 0.4803, 0.04741, 0.3222, 0.04491, 0.9911. From youngest to oldest, RR vs IR (suprabasal): p-values = 0.4840, 0.6448, 0.007985, 0.01514, 0.04887, 0.01623, 0.007354, 0.722, 0.7963. **(d)** Summary diagram of proliferative patterning in basal bud formation versus rete ridge formation. **(e)** Schematic outlining the BrdU-injection schedule and potential outcomes from the BrdU-labeling. **(f)** Representative immunostains from P8 (1 day post injection, 1dpi, n=3), P12 (5dpi, n=3), P16 (9dpi, n=3), and P16 negative control (-Ctrl, n=1) for BrdU/MKI67. Scale bars represent 100 um. **(g)** Stacked bar plots representing the proportion of all BrdU+ cells grouped by their layer of residence by timepoint. N=number of biological replicates, ir=number of inter-ridge regions quantified, rr=number of rete ridge

regions quantified. RR vs IR number of BrdU+ cells in the basal, suprabasal, spinous, and granular layers at P8 (p-values=0.1505, 0.09234, 0.2487, 0.6760), P12 (p-values=0.3392, 0.2385, 0.08829, 0.1465), P16 (0.5536, 0.5441, 0.2293, 0.9879) were not significant, p-values > 0.05 from t-test. **(h)** Quantification of proportion of BrdU+ keratinocytes per epidermal proliferation unit (EPU). RR vs IR at P8, P12, P16 not significant, p-values = 0.7659, 0.2547, 0.8920 from t-test. **(i)** Summary model of rete ridge and inter-ridge differentiation trajectories during rete ridge formation.

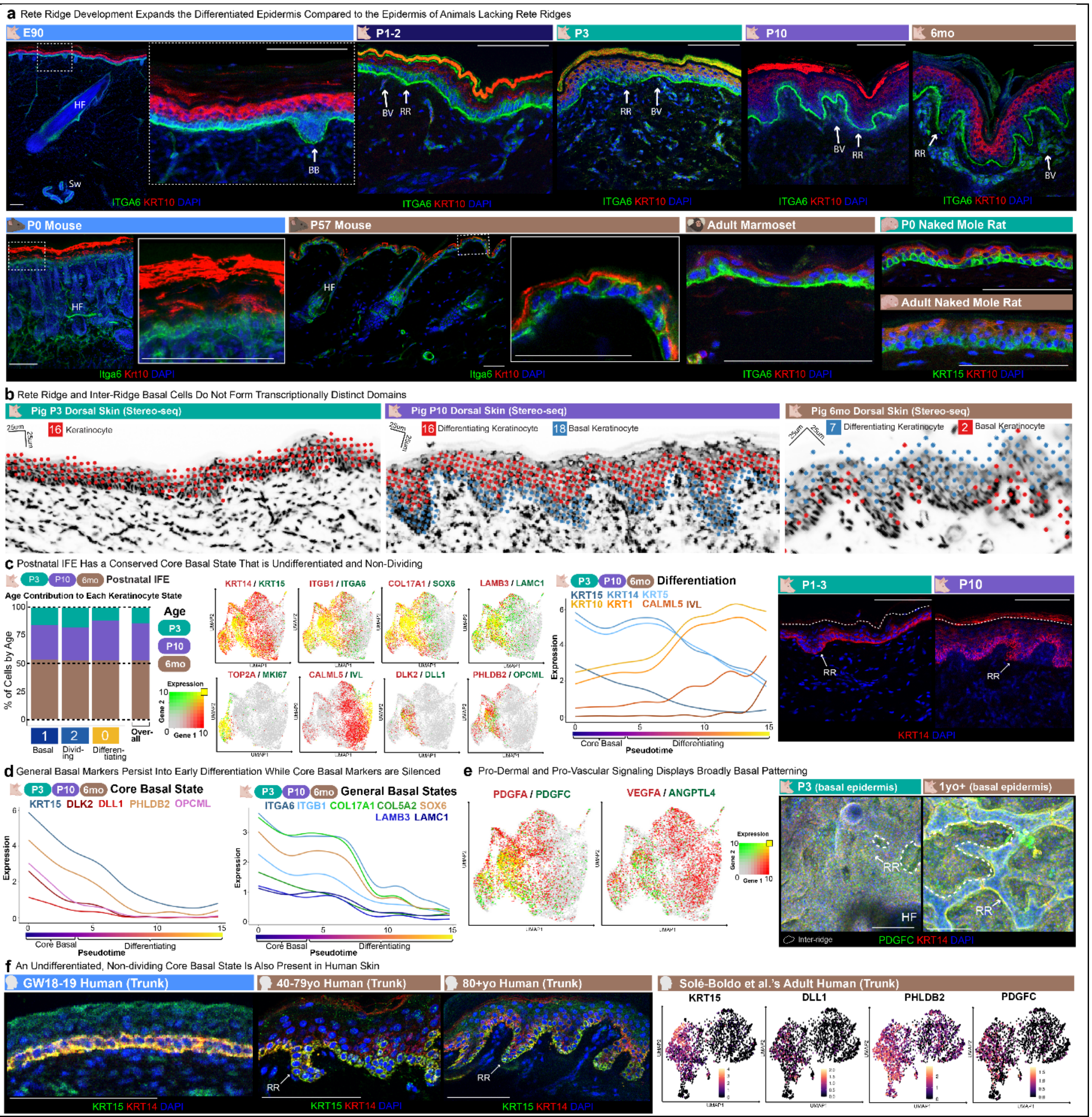

Supplemental Figure 7: A Core Basal State Defines Undifferentiating, Non-Dividing Basal Cells in Both the Rete Ridge and Inter-Ridge. (a) Representative immunostains for ITGA6/KRT10 from E90 (n=3), P1-2 (n=3), P3 (n=3), P10 (n=2), and 6mo (n=3) pig skin (top), P0 (n=3) and P57 (n=3) mice (bottom left), adult marmosets (n=1, bottom middle), and KRT15/KRT10 from neonatal (n=3) and adult (n=3) naked mole rats (bottom right). BB = basal bud/epithelial placode, RR = rete ridge, HF = hair follicle, Sw = sweat gland, BV = blood vessel. **(b)** Representative frames of interfollicular epidermis (IFE) from porcine P3, P10, 6mo Stereo-seq visualizing epidermal Leiden clusters. **(c)** Stacked bar plots representing the proportion of P3, P10, and 6mo IFE keratinocytes to each integrated cluster from scRNA-seq (from integrated P3-P10-6mo pig scRNA-seq IFE UMAP in Fig. 4c) (left). FeaturePlots depicting co-expression of canonical markers for basal states (KRT14, KRT15, ITGB1, ITGA6, COL17A1, SOX6, LAMB3, LAMC1), dividing states (TOP2A, MKI67), differentiation states (CALML5, IVL), and

differentially expressed genes defining an undifferentiated, non-dividing core basal state: DLK2, DLL1, PHLDB2, OPCML. Pseudotime trajectories for expression of canonical basal versus differentiation markers visualized as a LinePlot (from pseudotime UMAP Fig. 4c, e), and immunostaining validation of KRT14 core basal and suprabasal distribution in P1-3 (n=3) and P10 (n=2) pig skin. Dashed line indicates epidermal apical surface. **(d)** Pseudotime trajectories depicting expression of Core Basal State markers (KRT15, DLK2, DLL1, PHLDB2, OPCML) compared to General Basal Markers (ITGA6, ITGB1, COL17A1, COL5A2, SOX6, LAMB3, LAMC1) visualized as LinePlots. **(e)** Co-expression FeaturePlots (left) for PDGFA/PDGFC and VEGFA/ANGPTL4 in the integrated P3-P10-6mo pig scRNA-seq IFE. Yellow expression indicates co-expression of both genes. (right) epidermal wholemount immunostaining of PDGFC and KRT14 in P3 (n=3) and 1yo+ (n=2) pig skin. **(f)** Representative immunostaining of human trunk skin from GW18-19 (n=4), 40-79yo (n=4), and 80+yo (n=1) trunk skin for KRT15 / KRT14 (left). FeaturePlots depicting KRT15, DLL1, PHLDB2, and PDGFC expression in in Solé-Boldo et al. 2020's Adult Human Trunk Skin scRNA-seq epidermal subset (right). (a, c-f) Scale bars represent 100um.

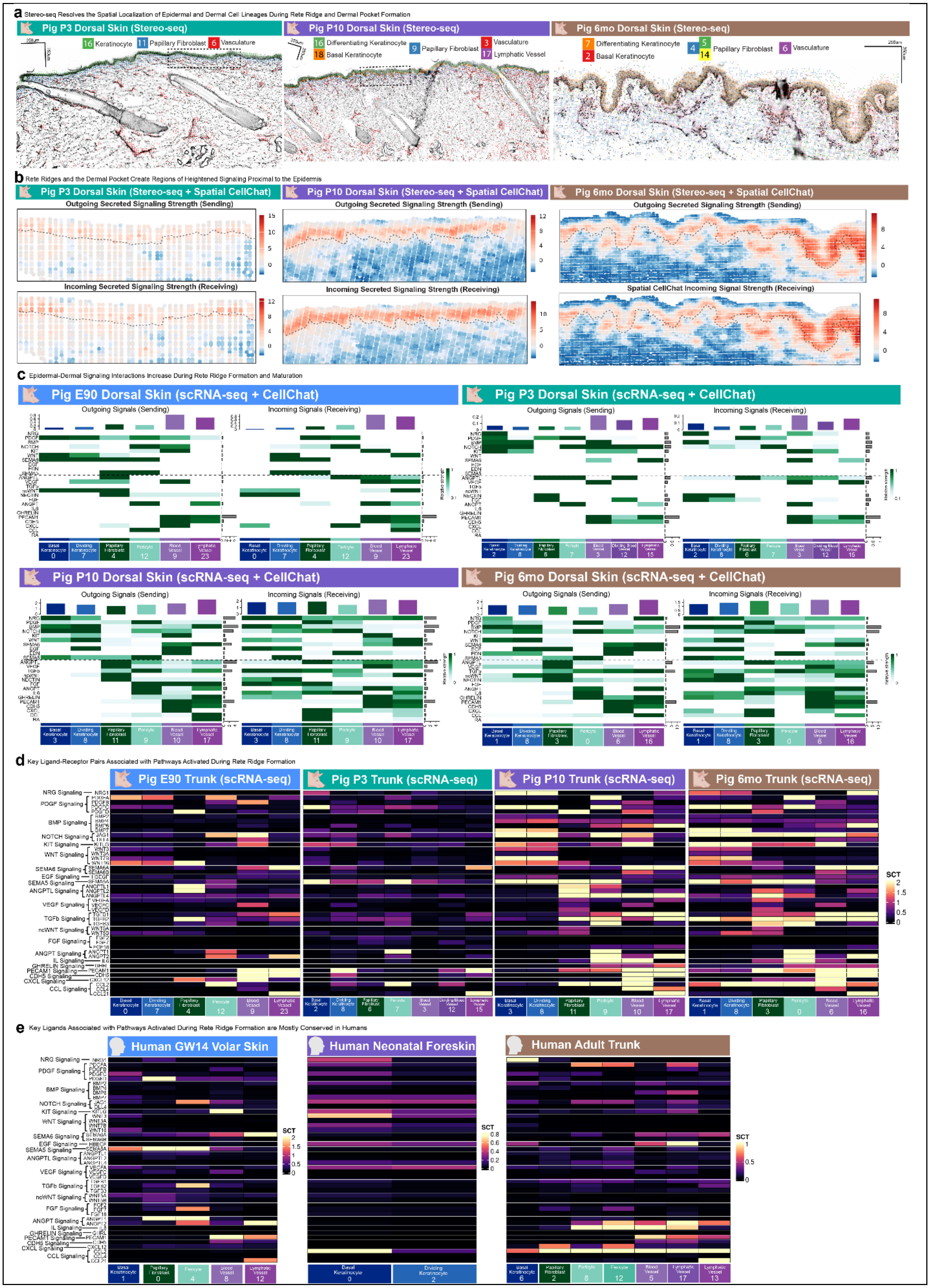
a Stereo-seq Resolves the Spatial Localization of Epidermal and Dermal Cell Lineages During Rete Ridge and Dermal Pocket Formation
Pig P3 Dorsal Skin (Stereo-seq)
Pig P10 Dorsal Skin (Stereo-seq)
Pig 6mo Dorsal Skin (Stereo-seq)
Keratinocyte
Papillary Fibroblast
Vasculature
Differentiating Keratinocyte
Basal Keratinocyte
Lymphatic Vessel
b Rete Ridges and the Dermal Pocket Create Regions of Heightened Signaling Proximal to the Epidermis
Pig P3 Dorsal Skin (Stereo-seq + Spatial CellChat)
Pig P10 Dorsal Skin (Stereo-seq + Spatial CellChat)
Pig 6mo Dorsal Skin (Stereo-seq + Spatial CellChat)
Outgoing Secreted Signaling Strength (Sending)
Incoming Secreted Signaling Strength (Receiving)
Spatial CellChat Incoming Signal Strength (Receiving)
c Epidermal-Dermal Signaling Interactions Increase During Rete Ridge Formation and Maturation
Pig E90 Dorsal Skin (scRNA-seq + CellChat)
Pig P3 Dorsal Skin (scRNA-seq + CellChat)
Pig P10 Dorsal Skin (scRNA-seq + CellChat)
Pig 6mo Dorsal Skin (scRNA-seq + CellChat)
Outgoing Signals (Sending)
Incoming Signals (Receiving)
d Key Ligand-Receptor Pairs Associated with Pathways Activated During Rete Ridge Formation
Pig E90 Trunk (scRNA-seq)
Pig P3 Trunk (scRNA-seq)
Pig P10 Trunk (scRNA-seq)
Pig 6mo Trunk (scRNA-seq)
NRG Signaling
PDGF Signaling
BMP Signaling
NOTCH Signaling
KIT Signaling
WNT Signaling
SEMA6 Signaling
EGF Signaling
SEMA5 Signaling
ANGPTL Signaling
VEGF Signaling
TGFb Signaling
ncWNT Signaling
FGF Signaling
ANGPT Signaling
IL Signaling
GHRELIN Signaling
PECAM1 Signaling
CDH5 Signaling
CXCL Signaling
CCL Signaling
SCT
e Key Ligands Associated with Pathways Activated During Rete Ridge Formation are Mostly Conserved in Humans
Human GW14 Volar Skin
Human Neonatal Foreskin
Human Adult Trunk
Basal Keratinocyte
Dividing Keratinocyte
Papillary Fibroblast
Pericyte
Blood Vessel
Lymphatic Vessel

Supplemental Figure 8: Spatial and Single-cell Transcriptomics Identify Signaling Pathways Activated During Rete Ridge Formation. (a) Stereo-seq data Leiden clusters for epidermal and dermal cell lineages projected over the tissue mask for P3, P10, and 6mo skin. Dashed boxes depict the zoomed in frames depicted in Fig. 4b. **(b)** Representative regions, as in (a), representing the overall outgoing and incoming communication score strength from Spatial CellChat on all clusters from P3, P10, and 6mo Stereo-seq data. Dashed line approximates the epidermal-dermal junction traced from the Stereo-seq Leiden-clustered image mask in (a). **(c)** Summary pathway-level heatmaps representing scRNA-seq clusters' sending (outgoing) and receiving (incoming) communication score strength, from CellChat on E90, P3, P10, and 6mo scRNA-seq data: NRG, PDGF, BMP, NOTCH, KIT, WNT, SEMA6, EGF, EDN, SEMA5, ANGPTL, VEGF, TGFb, ncWNT, NECTIN, FGF, ANGPT, IL6, GHRELIN, PECAM1, CDH5, CXCL, CCL, RA pathways. **(d)** Visualization of ligands for pathways from (c) implicated by porcine scRNA-seq CellChat across E90, P3, P10, and 6mo epidermal and dermal cell types. **(e)** Visualization of ligands for pathways implicated by porcine scRNA-seq CellChat across Glover et al. 2023's Human GW14 Volar Skin, Cheng et al. 2018's Human Neonatal Foreskin, and Solé-Boldo et al. 2020's Human Adult Trunk Skin scRNA-seq datasets.

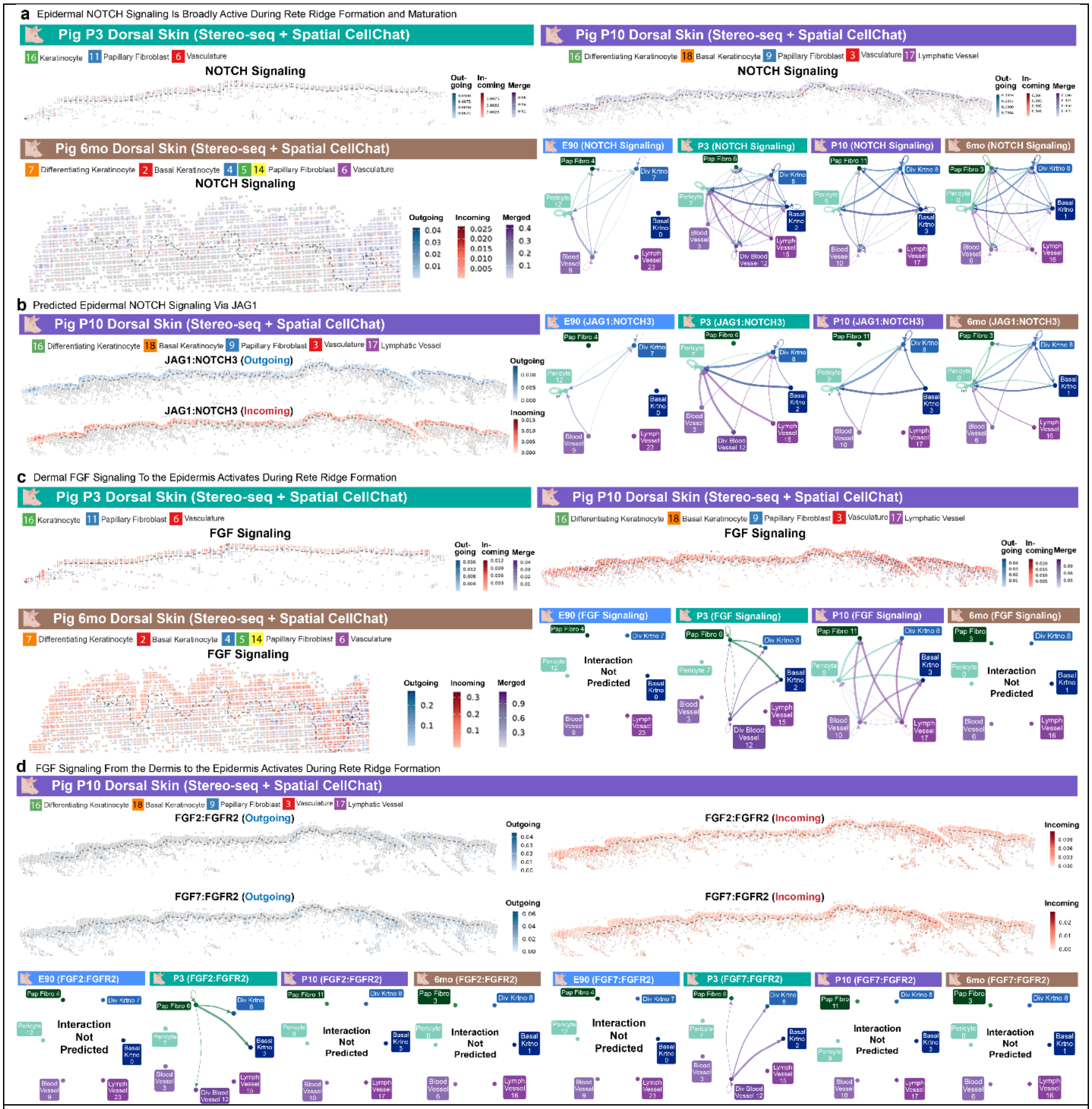

Supplemental Figure 9: Epidermal NOTCH and Dermal FGF Signaling Activate During Rete Ridge Formation. (a) Spatial CellChat outgoing/incoming communication score across the Stereo-seq data at P3, P10, and 6mo or CellChat CirclePlots for predicted signaling interactions from scRNA-seq data at E90, P3, P10, and 6mo for the NOTCH signaling pathway. **(b)** Spatial CellChat outgoing/incoming communication score across the Stereo-seq data at P3 and P10 or CellChat CirclePlots for predicted signaling interactions from scRNA-seq data at E90, P3, P10, and 6mo for NOTCH signaling ligand-receptor pairs. **(c)** Spatial CellChat outgoing/incoming communication score across the Stereo-seq data at P3, P10, and 6mo or CellChat CirclePlots for predicted signaling interactions from scRNA-seq data at E90, P3, P10, and 6mo for the FGF signaling pathway. **(d)** Spatial CellChat outgoing/incoming communication score across the Stereo-seq data at P10 or CellChat CirclePlots for predicted signaling interactions from scRNA-seq data at E90, P3, P10, and 6mo for FGF signaling ligand-receptor pairs. (a-d) Dashed line approximates the epidermal-dermal junction traced

from the Stereo-seq Leiden-clustered image mask (Supplemental Fig. 8a). Stereo-seq subset utilized in Spatial CellChat analyses consisted of epidermal keratinocytes, papillary fibroblasts, and vascular/pericyte clusters.

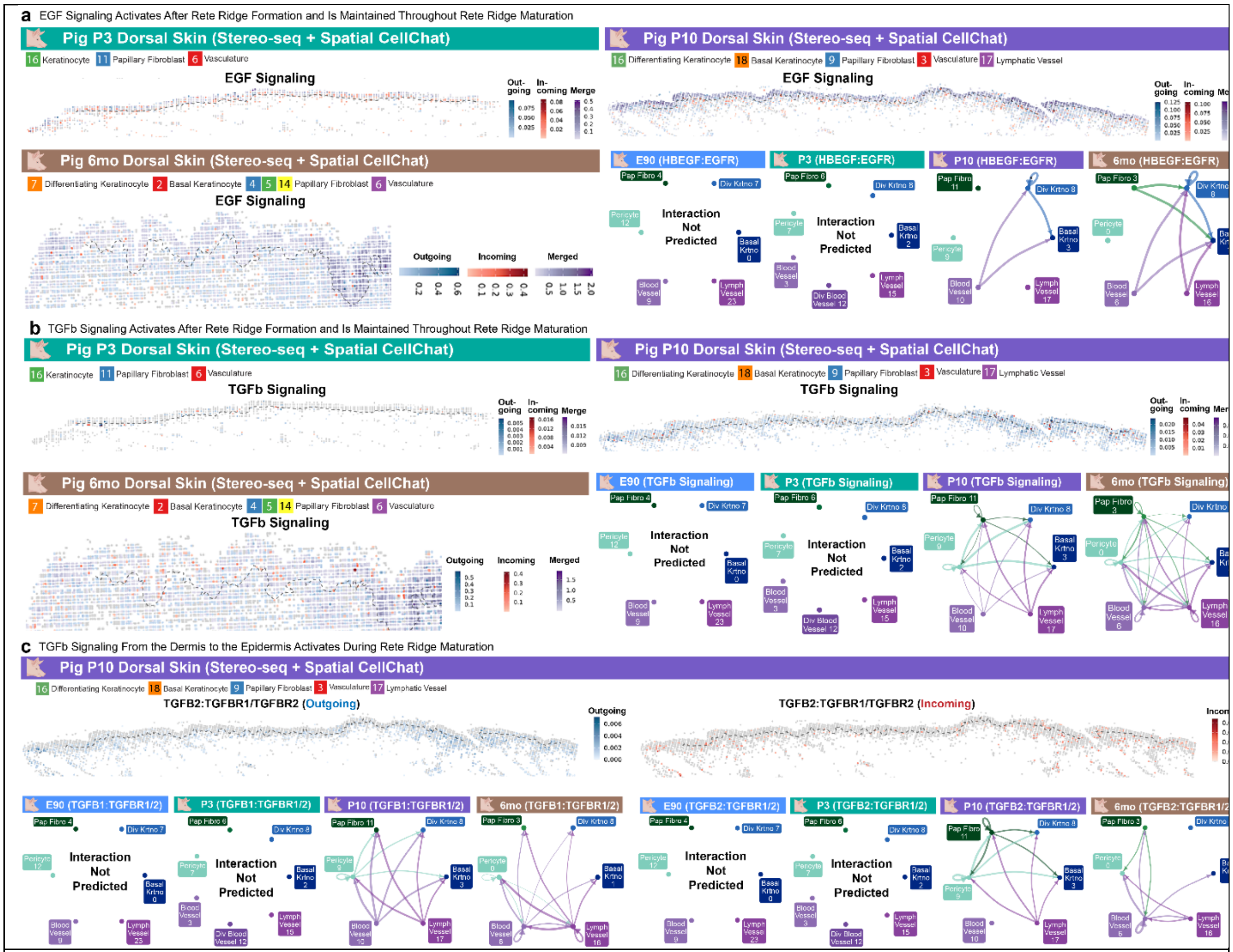

Supplemental Figure 10: EGF and TGFb Signaling Activation is Associated with the Transition from Rete Ridge Formation to Rete Ridge Maturation. (a) Spatial CellChat outgoing/incoming communication score across the Stereo-seq data at P3, P10, and 6mo or CellChat CirclePlots for predicted signaling interactions from scRNA-seq data at E90, P3, P10, and 6mo for EGF signaling ligand-receptor pairs. **(b)** Spatial CellChat outgoing/incoming communication score across the Stereo-seq data at P3, P10, and 6mo or CellChat CirclePlots for predicted signaling interactions from scRNA-seq data at E90, P3, P10, and 6mo for the TGFb signaling pathway. **(c)** Spatial CellChat outgoing/incoming communication score across the Stereo-seq data at P10 or CellChat CirclePlots for predicted signaling interactions from scRNA-seq data at E90, P3, P10, and 6mo for TGFb signaling ligand-receptor pairs. (a-c) Dashed line approximates the epidermal-dermal junction traced from the Stereo-seq Leiden-clustered image mask (Supplemental Fig. 8a). Stereo-seq subset utilized in Spatial CellChat analyses consisted of epidermal keratinocytes, papillary fibroblasts, and vascular/pericyte clusters.

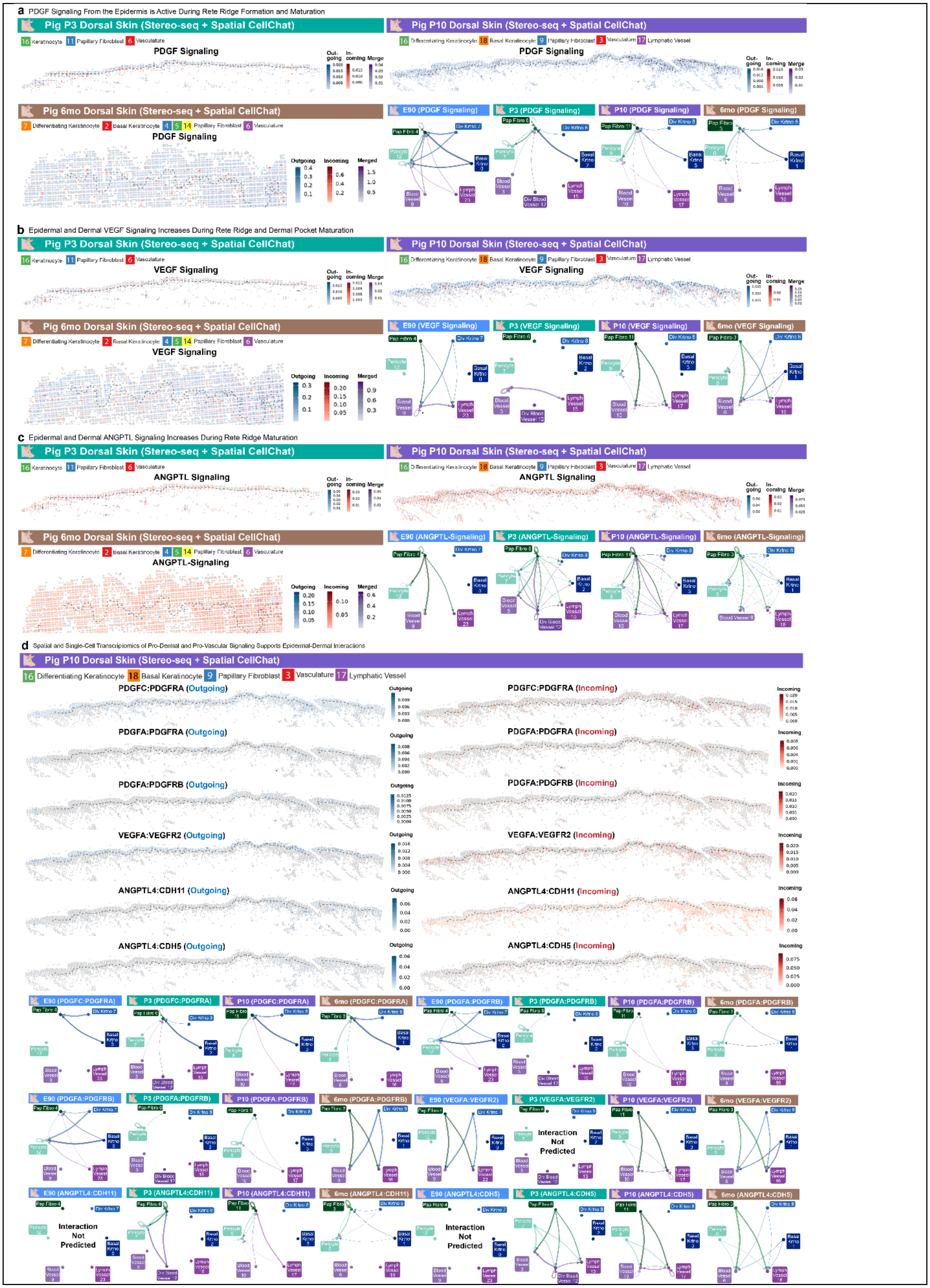
a PDGF Signaling From the Epidermis is Active During Rete Ridge Formation and Maturation
Pig P3 Dorsal Skin (Stereo-seq + Spatial CellChat)
Pig P10 Dorsal Skin (Stereo-seq + Spatial CellChat)
PDGF Signaling
Pig 6mo Dorsal Skin (Stereo-seq + Spatial CellChat)
b Epidermal and Dermal VEGF Signaling Increases During Rete Ridge and Dermal Pocket Maturation
VEGF Signaling
c Epidermal and Dermal ANGPTL Signaling Increases During Rete Ridge Maturation
ANGPTL Signaling
d Spatial and Single-Cell Transcriptomics of Pro-Dermal and Pro-Vascular Signaling Supports Epidermal-Dermal Interactions
PDGFC:PDGFRA (Outgoing)
PDGFC:PDGFRA (Incoming)
PDGFA:PDGFRA (Outgoing)
PDGFA:PDGFRA (Incoming)
PDGFA:PDGFRB (Outgoing)
PDGFA:PDGFRB (Incoming)
VEGFA:VEGFR2 (Outgoing)
VEGFA:VEGFR2 (Incoming)
ANGPTL4:CDH11 (Outgoing)
ANGPTL4:CDH11 (Incoming)
ANGPTL4:CDH5 (Outgoing)
ANGPTL4:CDH5 (Incoming)
Interaction Not Predicted

Supplemental Figure 11: Pro-dermal Signaling Increases Over the Course of Rete Ridge Formation and Maturation. (a) Spatial CellChat outgoing/incoming communication score across the Stereo-seq data at P3, P10, and 6mo or CellChat CirclePlots for predicted signaling interactions from scRNA-seq data at E90, P3, P10, and 6mo for the PDGF signaling pathway. **(b)** Spatial CellChat outgoing/incoming communication score across the Stereo-seq data at P3, P10, and 6mo or CellChat CirclePlots for predicted signaling interactions from scRNA-seq data at E90, P3, P10, and 6mo for the VEGF signaling pathway. **(c)** Spatial CellChat outgoing/incoming communication score across the Stereo-seq data at P3, P10, and 6mo or CellChat CirclePlots for predicted signaling interactions from scRNA-seq data at E90, P3, P10, and 6mo for the ANGPTL signaling pathway. **(d)** Spatial CellChat outgoing/incoming communication score across the Stereo-seq data at P10 or CellChat CirclePlots for predicted signaling interactions from scRNA-seq data at E90, P3, P10, and 6mo for PDGF, VEGF, and ANGPTL signaling ligand-receptor pairs. (a-d) Dashed line approximates the epidermal-dermal junction traced from the Stereo-seq Leiden-clustered image mask (Supplemental Fig. 8a). Stereo-seq subset utilized in Spatial CellChat analyses consisted of epidermal keratinocytes, papillary fibroblasts, and vascular/pericyte clusters.

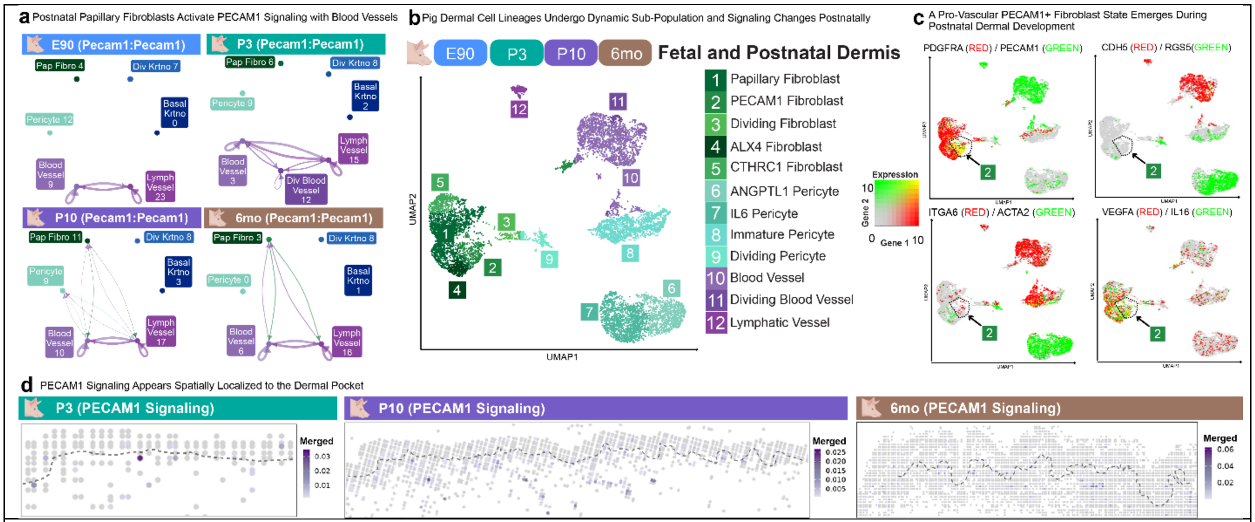

Supplemental Figure 12: A PECAM1+ Papillary Fibroblast Population Arises in the Dermal Pocket in Postnatal Pig Skin. (a) CellChat CirclePlots visualizing PECAM1 signaling between epidermal and dermal cell lineages from E90, P3, P10, and 6mo scRNA-seq datasets. **(b)** Integration of E90, P3, P10, and 6mo scRNA-seq dermal cell lineages visualized using UMAP. **(c)** Visualization of co-expression of marker genes for dermal cell lineages and signaling activities reveals a PDGFRA+/PECAM1+/ITGA6+/CDH5- fibroblast subpopulation. **(d)** Spatial context for PECAM1 signaling using Spatial CellChat from P3, P10, and 6mo Stereo-seq data. Dashed line approximates the epidermal-dermal junction traced from the Stereo-seq Leiden-clustered image mask (Supplemental Fig. 8a). Stereo-seq subset utilized in Spatial CellChat analyses consisted of epidermal keratinocytes, papillary fibroblasts, and vascular/pericyte clusters.

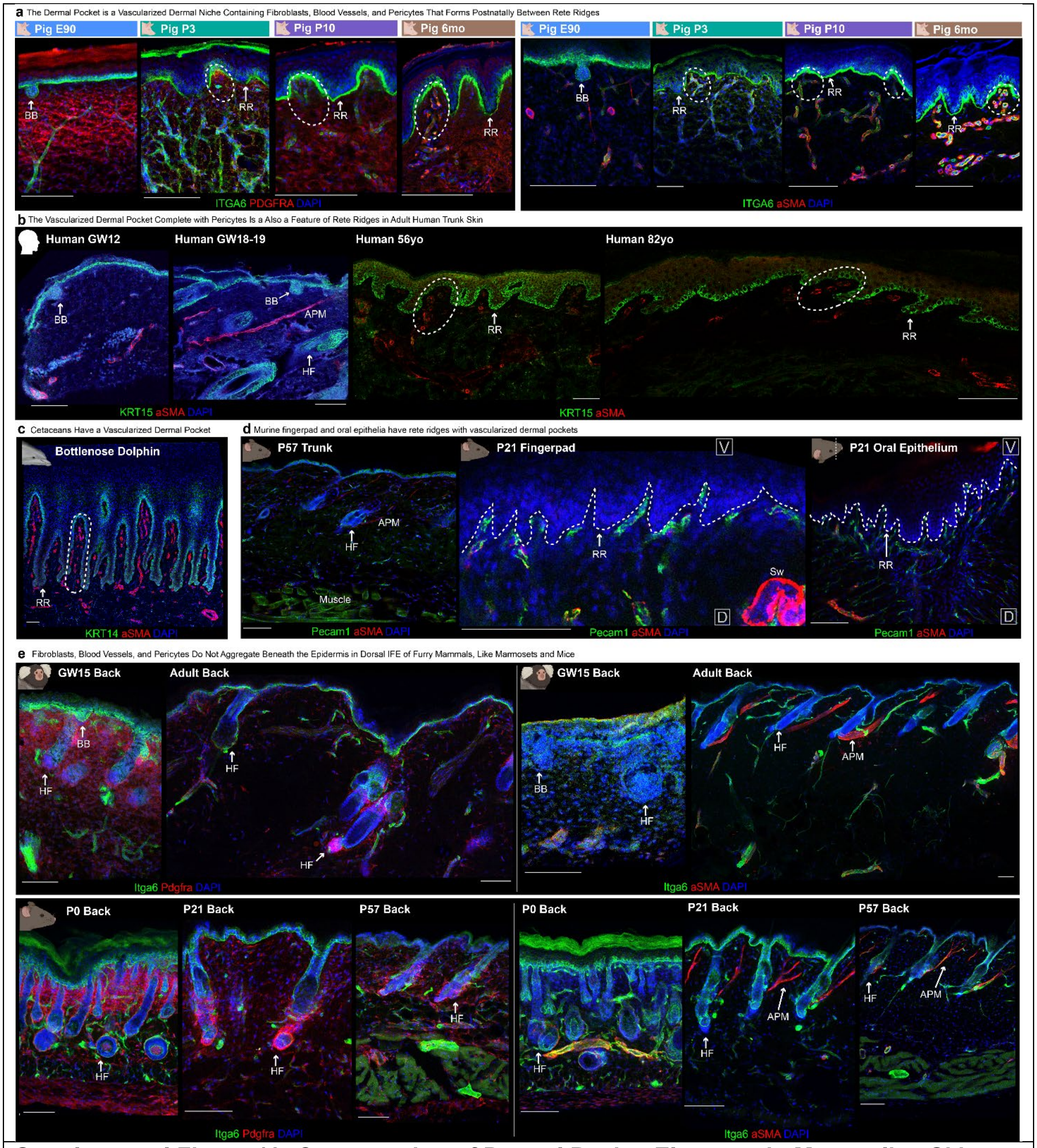

Supplemental Figure 13: Conservation of Dermal Pocket Elements in Mammalian Skin Containing Rete Ridges. (a) Representative immunostains of ITGA6/PDGFRA (left) and ITGA6/aSMA (right) from E90 (n=3), P3 (n=3), P10 (n=2), and 6mo (n=3) pig skin. Dashed ovals identify representative dermal pockets. BB = basal bud/epithelial placode, RR=rete ridge. **(b)** Representative immunostains of KRT15/aSMA to visualize pericyte localization across human skin development at GW12 (n=1), GW18-19 (n=2), 40-79yo (n=1), and 80+ yo (n=1). BB=basal bud, APM=arrector pili muscle, HF=hair follicle. **(c)** Representative immunostain of KRT14/aSMA to visualize pericyte localization in bottlenose dolphin skin (n=3). **(d)** Representative immunostain of vasculature and pericytes (PECAM1/aSMA) in (left to right) P57

mouse trunk (n=3), P21 mouse fingerpad (n=3), and P21 mouse oral epithelium (n=3). Boxed V and D indicate ventral-dorsal orientation of the tissue section. The epidermal-dermal junction is traced with a dashed line. **(e)** Representative immunostains of vasculature (Itga6), fibroblasts (Pdgfra), and pericytes (aSMA) across (top row) marmoset back skin development at GW15 (n=3) and adult (n=3) and (bottom row) mouse back skin development at P0 (n=3), P21 (n=3), and P57 (n=3). Scale bars in (a-e) represent 100um.
