# Supplementary material for: Rete ridges form via evolutionarily distinct mechanisms in mammalian skin
Source: Nature. 2026 Feb 4;651(8104):135–45. doi: 10.1038/s41586-025-10055-5 (PMC12959975; doi:10.1038/s41586-025-10055-5)
Supplement: Supplementary file 2 — Reporting Summary [file 41586_2025_10055_MOESM2_ESM.pdf]

nature portfolio

Corresponding author(s): Ryan R. Driskell Ph.D.

Last updated by author(s): Dec 4, 2025

Reporting Summary

Nature Portfolio wishes to improve the reproducibility of the work that we publish. This form provides structure for consistency and transparency in reporting. For further information on Nature Portfolio policies, see our Editorial Policies and the Editorial Policy Checklist.

Statistics

For all statistical analyses, confirm that the following items are present in the figure legend, table legend, main text, or Methods section.

n/a	Confirmed	
☐	☒	The exact sample size (*n*) for each experimental group/condition, given as a discrete number and unit of measurement
☐	☒	A statement on whether measurements were taken from distinct samples or whether the same sample was measured repeatedly
☐	☒	The statistical test(s) used AND whether they are one- or two-sided<br>*Only common tests should be described solely by name; describe more complex techniques in the Methods section.*
☒	☐	A description of all covariates tested
☐	☒	A description of any assumptions or corrections, such as tests of normality and adjustment for multiple comparisons
☐	☒	A full description of the statistical parameters including central tendency (e.g. means) or other basic estimates (e.g. regression coefficient) AND variation (e.g. standard deviation) or associated estimates of uncertainty (e.g. confidence intervals)
☒	☐	For null hypothesis testing, the test statistic (e.g. *F*, *t*, *r*) with confidence intervals, effect sizes, degrees of freedom and *P* value noted<br>*Give P values as exact values whenever suitable.*
☒	☐	For Bayesian analysis, information on the choice of priors and Markov chain Monte Carlo settings
☒	☐	For hierarchical and complex designs, identification of the appropriate level for tests and full reporting of outcomes
☒	☐	Estimates of effect sizes (e.g. Cohen's *d*, Pearson's *r*), indicating how they were calculated

Our web collection on statistics for biologists contains articles on many of the points above.

Software and code

Policy information about availability of computer code

Data collection	Image analysis and quantification was performed using Fiji ImageJ (v.1.53c). scRNA-seq data was generated from single-cell suspensions from E90, P3, P10, and 6mo pig skin using 10x Genomics scRNA-seq 3' V3 kit and sequenced on an Ilumina NovaSeq PE150 by Novogene. Stereo-seq data was generated from P3, P10, and 6mo fixed-frozen 10um cryo sections using Complete Genomics Stereo-seq T FF V1.2 kit and sequenced using a DNBSEQ-T7.
Data analysis	scRNA-seq Fastq files were aligned to the Sscrofa.11.1 genome assembly using 10x Genomics Cellranger (v6.0.0). Cellranger outputs were used in downstream analysis. Stereo-seq Fastq files were aligned to the Sscrofa.11.1.112 genome release from Ensembl using STOmics SAW (8.1.1) and SAW outputs were used in downstream analysis. Raw and processed data is deposited on GEO as described in our Methods. scRNA-seq data analysis was performed in R (v4.2.1) using the Seurat (v0.3.1), SeuratWrapper (v0.4.3), Monocle3 (v1.3.1), and CellChat (v1.4.0) packages. Statistical analyses of quantifications was analyzed in R (v4.2.2). Source code and more detailed software package and dependency versions is available on our Github page: https://github.com/DriskellLab/Thompson-et-al.-2025 . For Stereo-seq spatial transcriptomics, SAW (8.1.1) was utilized with Sscrofa11.1.112 release with Ensembl and makeRef Function. Tissue mask utilized Stereo Map 4. SAW realign Visualization output.gef files were loaded into and analyzed using Stereopy 1.5.0 in Python 3.8.20.<br>CellChat analysis was used to infer pathway and ligand-receptor interactions among core basal and dividing keratinocyte, papillary fibroblast, pericyte, and blood vessel clusters from porcine skin scRNA-seq datasets. Similarly, Spatial CellChat was used to infer pathway and ligand-receptor interactions from porcine Stereo-seq datasets.<br>Adobe Illustrator (v. 2021, 2023, 2025) and Adobe Photoshop (v. 2021, 2023, 2025) were used as described in the methods section.

For manuscripts utilizing custom algorithms or software that are central to the research but not yet described in published literature, software must be made available to editors and reviewers. We strongly encourage code deposition in a community repository (e.g. GitHub). See the Nature Portfolio guidelines for submitting code & software for further information.

Data

Policy information about availability of data

All manuscripts must include a data availability statement. This statement should provide the following information, where applicable:

- Accession codes, unique identifiers, or web links for publicly available datasets
- A description of any restrictions on data availability
- For clinical datasets or third party data, please ensure that the statement adheres to our policy

We have made all the analyzed scRNA-seq and Stereo-seq datasets in this study available via our interactive webtools on skinregeneration.org (https://skinregeneration.org/papers/Thompson-et-al-2025/). Raw and processed sequencing data files used in our analysis can be found at the NCBI's Gene Expression Omnibus (GEO accession number: GSE305111. Data underlying plots in this manuscript are fully available as Source Data, and exact p-values are provided in figure legends or in Source Data where there is insufficient space to include in the legend. All other data is available either as supplementary information or from the corresponding author upon reasonable request.

Research involving human participants, their data, or biological material

Policy information about studies with human participants or human data. See also policy information about sex, gender (identity/presentation), and sexual orientation and race, ethnicity and racism.

Reporting on sex and gender	All human tissue samples were obtained de-identified with basic age and sex data only. Both male and female samples were analyzed whenever possible.
Reporting on race, ethnicity, or other socially relevant groupings	Our samples were obtained de-identified without these types of data.
Population characteristics	Adult human tissues were obtained as surgical discard tissue from dermatology clinic procedures. Samples were grouped into the following categories 21-39 years old, 40-59 years old, 60-79 years old, and 80+ year old with most samples falling into the 60-79 years old category. Gestational human tissue samples ranging from gestational week 7 up to 20 were obtained by the University of Washington Birth Defects Research Laboratory following maternal consent. A mix of males and females were used in the study whenever possible.
Recruitment	Adult subjects were already undergoing a surgical procedure at Advanced Dermatology, Spokane, WA and surgical discard tissue was collected with informed consent in accordance with Washington State University IRB-approved protocols (Washington State University #19796). Gestational human tissue samples ranging from gestational week 7 up to 20 were obtained by the University of Washington Birth Defects Research Laboratory with maternal consent and in accordance with University of Washington IRB-approved protocols (University of Washington STUDY00000380). De-identified samples were received at Washington State University in accordance with Washington State University IRB-approved protocols (Washington State University #19680). Complete details are provided in methods.
Ethics oversight	Washington State University IRB (#19796, 19680), University of Washington IRB (STUDY00000380). Complete details are provided in methods.

Note that full information on the approval of the study protocol must also be provided in the manuscript.

Field-specific reporting

Please select the one below that is the best fit for your research. If you are not sure, read the appropriate sections before making your selection.

☒ Life sciences ☐ Behavioural & social sciences ☐ Ecological, evolutionary & environmental sciences

For a reference copy of the document with all sections, see nature.com/documents/nr-reporting-summary-flat.pdf

Life sciences study design

All studies must disclose on these points even when the disclosure is negative.

Sample size	No sample size calculations were performed. A minimum of 3 or more biological replicates, and a mix of males and females, were obtained when possible, however some timepoints within our human or pig sample sets only consisted of 1-2 biological replicates due to model limitations (e.g. initial pig litter size), which are clearly delineated in the figure legends. In mouse experiments, multiple litters and a mix of male and female individuals were used when possible with specifics included in the figure legends or methods section. In pig experiments, multiple litters were used when possible with specifics included in the figure legends or methods section. Sample sizes in this study are comparable to other studies in our field.
Data exclusions	No data was excluded
Replication	Sample collection consisted of 3 or more biological replicates when possible to ensure reproducibility within a given condition. Immunostaining was replicated by 3 or more biological replicates when possible to ensure consistency and reproducibility. Some timepoints

of human or pig samples only had 1-2 replicates due to difficulties in obtaining samples from these organisms, and these are explicitly stated in the Figure Legends or Methods sections. Experiments were repeated more than once when possible. Certain wounding experiments were only performed once but included multiple biological replicates. All replication attempts were successful when experiments were repeated. Complete details for each experiment are included in the figure legends as word limits permit or in the methods section.

Randomization

In mouse treatment experiments (tamoxifen induction of epidermal BMPR1A knockout), K14CreERT Bmpr1a fl/fl genotypes were treated with tamoxifen and used as "TAMX" (induced knockout) group while K14CreERT genotypes were treated with tamoxifen and used as control group (induced no knockout). For porcine wound healing and BrdU-labeling experiments, negative control animals and animals selected for collection at discrete timepoints (e.g. 28 days post wounding for wound experiment or 1 day post injection for BrdU-labeling) were chosen randomly.

Blinding

Researchers were not blinded to experimental conditions (e.g. genotype) when performing quantifications on images. Many quantifications in this manuscript were exploratory or temporal in nature (e.g. quantifying epidermal thickness or rete ridge density over developmental time) rather than comparisons between experimental groups, which has lower risk of subjective interpretation. For experimental comparisons, many phenotypes in transgenic mouse studies rendered it impossible to "blind" due to dramatic morphological differences: K14-Noggin and tamoxifen-treated K14CreERT Bmpr1a fl/fl mice have major digit tip phenotypes due to the role of BMP signaling in nail or phalanx development and homeostasis which make blinding genotype impossible compared to controls. Similarly Lef1-eKO back skin and digits are also impossible to blind due to their dramatic hair reduction. Similarly, EDA-KO pigs have a hair phenotype which is discernable in histology compared to age-matched wild-type pigs (Ostedgaard et al. 2020. Elife). Finally, statistical analyses were performed after full collection of data from experimental groups and controls. Statistical comparisons between species in Figure 2c were performed multiple times using identical statistical methodology (one-way ANOVA) over the course of the manuscript revisions since additional biological replicates for some species were added between the original submission and the final revision. Critically, all quantifications utilized multiple technical replicates (e.g. different tissue sections or different skin samples collected from the same organism) which were averaged to determine the value of each biological replicate. In all studies, one biological replicate represented one distinct animal. For porcine wound healing and BrdU-labeling experiments, the surgeon or injector, respectively, was blinded to the eventual collection timepoint of the animal.

Reporting for specific materials, systems and methods

We require information from authors about some types of materials, experimental systems and methods used in many studies. Here, indicate whether each material, system or method listed is relevant to your study. If you are not sure if a list item applies to your research, read the appropriate section before selecting a response.

Materials & experimental systems

n/a	Involved in the study
☐	☒ Antibodies
☒	☐ Eukaryotic cell lines
☒	☐ Palaeontology and archaeology
☐	☒ Animals and other organisms
☒	☐ Clinical data
☒	☐ Dual use research of concern
☒	☐ Plants

Methods

n/a	Involved in the study
☒	☐ ChIP-seq
☒	☐ Flow cytometry
☒	☐ MRI-based neuroimaging

Antibodies

Antibodies used

Human-ITGA6 rat (1:200, BD Biosciences, Catalog # 555735, clone GoH3, lot # 8136528, 3055226, 1214287, 1033227), human LEF1 rabbit (1:200, Cell Signaling, Catalog # 2230S, clone C12A5, lot # 8, 9), human-aSMA rabbit (1:1000, Abcam, Catalog # ab5694, clone proprietary, lot # 1038192-2), human-PDGFRA goat (1:250, R&D Systems, Catalog # AF307NA, clone P16234, lot # VG0721111) in pig and marmoset samples, mouse-PDGFRA goat (1:250, R&D Systems, Catalog # AF110, clone P26618, lot # HMQ0222061) in mouse samples, human-KRT10 rabbit (1:250, Dennis Roop), human-KRT15 chicken (1:250, BioLegend, Catalog # 833904, clone Poly18339, lot # B353424, B404683), mouse-SOX9 rabbit (1:1000, EMDMillipore, Catalog # AB5535, clone P48436 C-term, lot # 3677685), human-MKI67 rabbit (1:400, Cell Signaling, Catalog # 9129S, clone D3B5, lot # 3, 9), BrdU rat (1:200, Abcam, Catalog # ab6326, clone BU1/75 (ICR1), lot # 1009715-43, 1009715-48), human-KRT15 rabbit (1:200, Sigma, Catalog # HPA024554, clone APREST75794, lot # A119308), human-SMAD1 goat (1:200, R&D, Catalog # AF2039, clone Q15797, lot # KOE062503A), human-pSMAD1/5 rabbit (1:200, Cell Signaling, Catalog # 9516, clone 41D10, lot # 10), human-KRT14 rabbit (1:250, Dennis Roop), human-KRT14 mouse (1:1000, R&D, Catalog # mab3164, clone LL001, lot # WEY0823012), human-PDGFC goat (1:200, R&D, Catalog # af1650, clone Q9NRA1, lot # JDI022406A), mouse-PECAM1 rat (1:200, Thermofisher, Catalog # 12-0311-82, clone 390, lot # 1989060, 3095164), mouse-PDGFC rat (1:200, R&D, Catalog # mab1447, clone Q8CI19, lot # HYQ022406A). Secondary antibodies used were AlexaFluor (AF) 488 anti-rat (1:1000, Fisher, Catalog # A21208, clone AB_2535794, lot # 2482958, 2668657, 2180272), AF488 anti-chicken (1:1000, Fisher, Catalog # A11039, clone AB_2534096, lot # 1899514, 2941307), AF488 anti-rabbit (1:1000, Fisher, Catalog # A21206, clone AB_2535792, lot #1874771), AF Plus 555 anti-rabbit (1:1000, Fisher, Catalog # A32794, clone AB_2762834, lot # VK307588, VD297829), AF555 anti-rabbit (1:1000, Fisher, Catalog # A31572, clone AB_2535849, lot # 2831376, 2482963), AF555 anti-goat (1:1000, Fisher, Catalog # A21432, clone AB_2535853, lot # 1878842, 2400919), AF Plus 555 anti-rat (1:1000, Fisher, Catalog # A48270, clone AB_2896336, lot # WF333067, ZG398235), AF647 anti-rabbit (1:1000, Fisher, Catalog # A31573, clone AB_2536183, lot # 2544598), AF647 anti-goat (1:1000, Fisher, Catalog # A21447, clone AB_2535864, lot # 1841382, 2297623). DAPI 300uM stock (1:1000, BioLegend, Catalog # 422801, lot # B222486, B324682) was used alongside secondary antibodies.

Validation

We validated antibodies based on known protein localization from ours and previous studies:
Human-ITGA6 rat (BD Biosciences): manufacturer validated in flow cytometry of human, mouse, pig, and dog cells. We and others have previously utilized this antibody for immunofluorescence staining of tissue sections (DOIs: 10.1016/0014-4827(90)90277-h,

10.1177/34.8.2426332, 10.7554/eLife.60066). Human-LEF1 rabbit (Cell Signaling): manufacturer validated in western blotting, immunofluorescence, and flow cytometry of human, mouse, and rat cells. We and others have previously used this antibody for immunofluorescence staining of tissue sections (DOI: 10.7554/eLife.60066). Human-aSMA rabbit (Abcam): manufacturer validated in western blotting, immunofluorescence of human and mouse cells. We and others have previously utilized this antibody for immunofluorescence staining of tissue sections (DOI: 10.7554/eLife.60066). Human-PDGFRA goat (R&D): manufacturer validated in western blotting and immunohistochemistry of human cells (DOI: 10.1016/j.jid.2018.01.016). Mouse-PDGFRA goat (R&D): manufacturer validated in western blotting, flow cytometry, and immunohistochemistry. We and others have previously utilized this antibody for immunofluorescence staining of tissue sections (DOI: 10.7554/eLife.60066, 10.1016/j.jid.2018.01.016). human-KRT10 rabbit (Dennis Roop): previously utilized for skin cross section immunofluorescence (DOI:10.1242/jcs.03298). Human-KRT15 chicken (BioLegend): manufacturer validated for western blotting and immunohistochemistry and published immunofluorescence staining in mouse skin tissue sections (DOI: 10.1038/s41467-022-30800-y). Human-SOX9 rabbit (EMDMillipore): manufacturer validated for ChIP, immunocytochemistry, immunohistochemistry, immunofluorescence, and western blotting of chicken, human, rat, mouse, and predicted for bovine, sheep, feline, equine with some literature validation (DOI: 10.1095/biolreprod.116.139832, 10.1038/s41467-019-10596-0). Human-MKI67 rabbit (Cell Signaling): manufacturer validated immunofluorescence (frozen), immunofluorescence (immunocytochemistry), flow cytometry of human, mouse, and rat cells. We and others have previously utilized this antibody for immunofluorescence staining of tissue sections (DOI: 10.1016/j.devcel.2022.06.005). anti-BrdU rat (Abcam): manufacturer validated for immunohistochemistry, flow cytometry, immunofluorescence (immunocytochemistry) of incorporated BrdU (species agnostic). This antibody has previously been utilized to identify BrdU incorporation in skin cross sections (DOI: 10.1242/dev.064592). Human-KRT15 rabbit (Sigma): manufacturer validated for immunoblotting, immunofluorescence, and immunohistochemistry in humans (DOI: 10.1016/j.devcel.2020.01.033). Human-SMAD1 goat (R&D): manufacturer validated for western blotting and immunohistochemistry (DOI: 10.2337/db17/1043). Human pSMAD1/5 (Cell Signaling): manufacturer validated for western blotting, immunofluorescence (immunocytochemistry), and flow cytometry in humans, mice, and rats. This antibody has previously been used to stain skin sections (DOI: 10.1038/ncb3535; 10.1038/s41467-019-09402-8). Human-KRT14 rabbit (Dennis Roop): previously utilized for skin (DOI:10.1242/jcs.03298). Human-KRT14 mouse (R&D): manufacturer validated for western blotting, immunocytochemistry, immunohistochemistry in humans (DOI: 10.1016/j.devcel.2022.05.003). Human-PDGFC goat (R&D): manufacturer validated for western blotting and immunohistochemistry in humans. Previously utilized with human cell in vitro (DOI: 10.18632/oncotarget.18706). Mouse-PECAM1 rat (Thermofisher): manufacturer validated for flow cytometry in mouse and published in C. elegans, fish, hamster, human, and mouse (DOI: 10.1038/s41598-018-38366-w). Mouse-PDGFC rat (R&D): manufacturer validated for western blotting in mice and does not cross-react with human-PDGFC (DOI: 10.1074/jbc.M111.222513).

Animals and other research organisms

Policy information about studies involving animals; ARRIVE guidelines recommended for reporting animal research, and Sex and Gender in Research

Laboratory animals	Mice (C57BL/6 mixed background ranging in age from P0 to adults older than 1-year, housed in a climate controlled facility set to ~68-73 degrees Fahrenheit and ~40% humidity. TEMP and humidity, with a 12-hour light/dark cycle with food and water ad libitum). Naked Mole Rat (Heterocephalus glaber), Common Marmoset (Callithrix jacchus), North American Grizzly Bear (Ursus arctos horribilis), Rhesus Macaque (Macaca mulatta), Pig (Sus scrofa) housed in a climate controlled facility ranging ~70-80 degrees Fahrenheit and ~30-50% humidity in a 12-hour light/dark cycle.
Wild animals	The study did not involve wild animals.
Reporting on sex	Sex is indicated where applicable by colored dots in graph. We made every effort to include equal numbers of each sex when possible.
Field-collected samples	No field-collected samples were used in this study.
Ethics oversight	Adult naked mole rats (Heterocephalus glaber) were maintained at the University of Texas Health Science Center at San Antonio for unrelated studies performed under protocols approved by the University of Texas Health Science Center at San Antonio IACUC (#20210034AR).<br><br>Rhesus macaques (Macaca mulatta) were maintained at the Oregon National Primate Research Center (ONPRC) at Oregon Health and Science University for unrelated studies performed under protocols approved by Oregon Health and Science University IACUC (IP03716, IP03276, IP00367). Additionally, the ONPRC is accredited by the Association for Assessment and Accreditation of Laboratory Animal Care (AAALAC; Animal Welfare Assurance D16-00195) and registered with the USDA (#92-R-001). Rhesus macaque samples were shared through the ONPRC tissue distribution program.<br><br>Common marmosets (Callithrix jacchus) were maintained at the Southwest National Primate Research Center at Texas Biomedical Research Institute for unrelated studies performed under an approved animal use protocol (Assurance Number D16-00048).<br><br>North American grizzly bears (Ursus arctos horribilis) were housed at the Washington State University Bear Research, Education, and Conservation Center. Bears were anesthetized and biopsies collected for unrelated studies performed under Washington State University IACUC-approved protocols (#6546).<br><br>Mice (Mus musculus) used in this study were of a mixed wild-type C57BL/6 background housed at Washington State University. Mouse housing and skin sample collection were in accordance with Washington State University IACUC-approved protocols (#6723, 6724, 6930).<br><br>Gestational human tissue samples were obtained by the Birth Defects Research Laboratory at University of Washington under University of Washington IRB-approved protocols with maternal written consent (University of Washington STUDY00000380; Washington State University 19680).

Adult human tissue samples were obtained by Advanced Dermatology in Spokane, Washington as surgical discard tissue after informed consent and in accordance with Washington State University IRB approved protocols (19796). All human tissue samples were deidentified prior to receipt at Washington State University and were analyzed in accordance with Washington State University IRB-approved protocols (19680, 19796).

Pigs (Sus scrofa) were housed at Washington State University under approved IACUC and USDA protocols (6492). Some fetal pigs were obtained from Biology Products (Alexandria, MN) and were exempt from Washington State University IACUC approval. Embryonic day 90 fetal pigs were harvested post-mortem from a pregnant pig with known date of conception under WSU IACUC-approved protocols (6492). Postnatal pig skin samples were collected post-mortem from pigs either housed at Washington State University, obtained from local farmers, or collected post-mortem from local butchers in accordance with Washington State University IACUC-approved protocols (6492). Yucatan Hairless pig and Hanford mini pig tissue samples were received from Sinclair Biosciences (Auxvasse, MO) and Mangalitsa pig tissue samples were obtained following butchering and were exempt from Washington State University IACUC approval. EDA-KO pig samples were collected by the Welsh and Ostedgaard Labs at University of Iowa (Ostedgaard et al., 2020) under University of Iowa IACUC-approved protocols (3071121). All experiments followed relevant guidelines and regulations from the appropriate ethics committees, as detailed above.

Bottlenose dolphin (Tursiops truncatus), long-beaked common dolphin (Delphinus capensis), and short-beaked common dolphin (Delphinus delphis) skin used in this study were obtained by the Plikus Lab at the University of California, Irvine, from NOAA (Southwest Fisheries Science Center, La Jolla, California) under the destructive loan permit. The analyzed specimens include: Delphinus capensis (numbers KXD0225, KXD0226, 1741-2023 Dc2301B), Delphinus delphis (numbers BLH0012, KXD0357, 585-2022 Dd2202B), and Tursiops truncatus (numbers KXD0410, KZP0069, 812-2022 Tt2202B).

K14CreERT Bmpr1a fl/fl tamoxifen-treated and control mice used in this study were housed at the University of Warsaw. The animal studies were approved by the First Local Ethics Committee: No. 971/2020 as of 28 January 2020, No. 1669/2025 as of 18 March 2025.
3mo WT and K14-Noggin mice were analyzed from archived paraffin-embedded digit samples (Plikus et al. 2004) as described in the Methods, and no new animal experiments were performed.

Note that full information on the approval of the study protocol must also be provided in the manuscript.

Plants

Seed stocks	Not applicable.
Novel plant genotypes	Not applicable.
Authentication	Not applicable.
